# On the Treatment of Articular Rheumatism, by Tincture of Colchicum, Nitre and Blood-letting

**Published:** 1845-01

**Authors:** 


*On the Treatment of Articular Rheumatism, by Tincture of Colchicum, Nitre and Blood-letting.*—Dr. E. MONNERET has made some interesting observations on these points of practice, which deserve the serious consideration of the profession. Having shown, in a preceding paper, that the sulphate of quinine had no title to rank as a therapeutical agent in rheumatism of efficacy superior to many others, he now proceeds to test the value of the articles indicated above.

Twenty-five patients were treated by the tincture of the colchicum root, eight by nitre, and nineteen by copious blood-letting.

The energy of the preparation of colchicum was first ascertained—it was very powerful. The great number of the patients took from four to sixteen grammes (one drachm to four drachms) in the course of the twenty-four hours, in one, two, or four divided doses. No smaller dose than a drachm was ever administered, and several of the patients took it for seven, some for ten, and others for thirteen days. The medicine was not discontinued in any case until it was ascertained to have no effect upon the disease.

In eight of the patients, the diminution, or even total disappearance of the symptoms of rheumatism, coincided with the exhibition of the tincture of colchicum. The rheumatism in these cases was either of some days' duration, and was scarcely accompanied with febrile symptoms, and then ended in twelve or fourteen days, or it was completely chronic. In either case the powerful revulsion produced by the tincture of colchicum on the bowels sufficed to suspend or to expel the disease: the improvement always coincided with the diarrhœa. In no case did the tincture of colchicum produce amendment or cure of rheumatism by any of those specific and occult properties which have been recognized in it by certain writers. In a few rare cases where its action was beneficial and rapid, it operated as a true drastic purgative. With regard to any complications which existed with the disease on the side of the heart, M. Monneret observes that it is scarcely needful for him to say that they were in nowise modified by the tincture of colchicum. If the effects of this medicine upon rheumatism, then, are nil in fact, which seems quite certain, it is much otherwise in so far as the abdominal viscera are concerned. Upon this point considerable difference of opinion appears to prevail: some say the colchicum occasions no intestinal disturbance, and that it does not purge; others maintain that it abates the pulse in force and frequency; and almost all unite in lauding its effects in rheumatism, &c. I have watched its influence in a sufficient number of instances, says M. Monneret, to have no hesitation in stating exactly what I have seen. In twenty-five patients to whom the tincture of colchicum was administered, I observed but a single order of phenomena at all referable to the gastro-intestinal system. The most remarkable among them were nausea and vomiting, diarrhœa, colic pains, and borborygmi, and the whole of these effects almost immediately followed the exhibition of the medicine in large doses, and for a certain time. In other instances, diarrhœa was the prevailing feature—there was little sickness or vomiting, but the alvine evacuations were copious and repeated. In a third and very small class the chief complaint was of nausea and vomiting without any purging.

The sickness supervened either immediately after taking the draught, or at some longer or shorter interval during the day or night. The discharges were almost always bilious, or evidently mixed with bile. The diarrhœa was generally in proportion to the dose; from one to two drachms of the tincture were followed by from two to twenty evacuations in the course of the twenty-four hours. The motions were mostly passed with acute suffering, violent colic pains in the bowels, tenesmus, and scalding of the anus. The matters passed were at first semi-fluid, but by and by they consisted in great part of a yellow and evidently bilious serum, in which floated a large quantity of whitish grains in form and color like the ova of a fish's roe; there was also mixed with them a quantity of red matter like scrapings of meat, and some blood more or less mixed with mucus.

Vomiting was scarcely induced by a smaller dose of the tincture than from two to four drachms in a draught; it will not follow one drachm, two drachms, or even three drachms administered in a large quantity of tisan. Several elements enter into the consideration of the therapeutical effects of medicines: the dose, the mode of administration, and the intervals of repetition. The effects of remedies are signally different from those generally seen when they are given in large and closely repeated doses. Three drachms of tincture of colchicum in two doses, one close upon the other, produce effects which are not only more energetic, but also different from those generally witnessed.

It is obvious, therefore, that colchicum in tincture exerts its agency especially upon the bowels. Of what nature is this agency? The diarrhœa, the dysenterical character of the stools, the severe griping, which follows its exhibition, do not continue as in cases in which the intestinal mucous membrane is truly inflamed; its effect is mainly to alter the secreting faculty of the intestines—the fluids habitually poured out are increased in quantity, and changed in quality.

Colchicum appears to have no effect upon the urinary secretions; and must, therefore, be rased from the number of diuretics.

*Blood-letting.*—In nineteen cases of acute articular rheumatism, desiring to ascertain the effects of a somewhat energetic antiphlogistic treatment, the patients were bled at least three times each in the course of the four first days, and cupping was further had recourse to around the affected joints, or to the region of the heart: only in two of the cases were tartar emetic and digitalis exhibited simultaneously. The quantity of blood abstracted was considerable,—large; and the venesections were repeated at short intervals. The mean stay in hospital of the patients thus treated was fourteen days—about the same as when other plans of treatment were employed.

The effect of the blood-letting on the disease can always be judged of by the state of the pulse: if it becomes less frequent, and loses force and volume, and if the temperature of the surface at the same time declines, the disease will end; if the pulse continues frequent, the disease is not yet at its conclusion. Sometimes the pulse falls suddenly after the first or second bleeding and the disease appears about to be subdued; but it soon rises again to its old number, and matters go on as if there had been no prospect of amendment: the gradual and enduring fall of the pulse is the best sign of improvement; if it falls from six to twelve beats below its usual number, so much the better.

When the symptoms are not relieved by the blood-letting within the first four or five days of the invasion of the disease, it is vain persisting in the abstraction of blood; the practice then is only injurious; bellows murmurs are set up in the heart and great vessels, the surface becomes drenched in sweat, the sleep is disturbed, the pulse is rapid, and the pains, far from diminishing, flirt about from one joint to another, or remain obstinately fixed in those that were first attacked. The conclusion, on the whole, in regard to blood-letting is that in moderation it is useful, especially when practised early in the disease, within the first four days; after this, depletion by the lancet only reduces the patient, and renders his recovery more difficult.

*Nitre.*—Eight patients only were treated with nitre, and of the number one was affected with meningitis cerebro-spinalis, another with pneumonia. In all the rheumatism was recent and severe. The medicine was administered in doses of from eight to thirty grammes (two to seven and a half drachms) dissolved in tisan. Its influence appeared to be absolutely nil in the whole of the cases. The pains in the joints, the signs of endocarditis, underwent no kind of diminution under its influence. The pulse was not lowered, the febrile heat was not lessened by it. The quantity of urine passed in the twenty-four hours was not increased. In order to control the disease it was necessary, in every case, to have recourse to other means.—*London Med. Gaz.*, from *Archives Generales, in American Journal.*

